# Card of the Committee on Prize Essays of the American Medical Association

**Published:** 1855-12

**Authors:** 


*Card of the Committee on Prize Essays of the American Medical Association.*—At a meeting of the American Medical Association, held in Philadelphia, May, 1855, the undersigned were appointed a committee to receive voluntary communications on Medical Subjects, and to award prizes in accordance with regulations of that body.

Each communication intended to compete for a prize, must be addressed to the Chairman of the Committee, at Ann Arbor, Michigan, before March 20th, 1856, and must be accompanied with a sealed packet containing the name of the author, and marked anteriorly by a sentence or motto corresponding with one upon the essay belonging to it; the packet to remain unopened unless is successful in obtaining a prize.

Unsuccessful papers will be returned, on application, after the adjournment of the association, at Detroit, in May next.

A. B. Palmer, M. D., Chairman.
S. Denton, M. D.,
A. R. Terry, M. D.,
A. Sager, M. D.,
S. H. Douglass, M. D.,
C. L. Ford, M. D.,
E. Andrews, M. D.

